# Effective integration of multi-omics with prior knowledge to identify biomarkers via explainable graph neural networks

**DOI:** 10.1038/s41540-025-00519-9

**Published:** 2025-05-08

**Authors:** Rohit K. Tripathy, Zachary Frohock, Hong Wang, Gregory A. Cary, Stephen Keegan, Gregory W. Carter, Yi Li

**Affiliations:** 1 The Jackson Laboratory for Genomic Medicine, Farmington, CT USA; 2 The Jackson Laboratory, Bar Harbor, ME USA

**Keywords:** Computational biology and bioinformatics, Molecular biology

## Abstract

The rapid growth of multi-omics datasets and the wealth of biological knowledge necessitates the development of effective methods for their integration. Such methods are essential for building predictive models and identifying drug targets based on a limited number of samples. We propose a framework called GNNRAI for the supervised integration of multi-omics data with biological priors represented as knowledge graphs. Our framework leverages graph neural networks (GNNs) to model the correlation structures among features from high-dimensional ‘omics data, which reduces the effective dimensions in data and enables us to analyze thousands of genes simultaneously using hundreds of samples. Furthermore, our framework incorporates explainability methods to elucidate informative biomarkers. We apply our framework to Alzheimer’s disease (AD) multi-omics data, showing that the integration of transcriptomics and proteomics data with prior AD knowledge is effective, improving the prediction accuracy of AD status over single-omics analyses and highlighting both known and novel AD-predictive biomarkers.

## Introduction

Advances in high-throughput technologies have led to an explosion in the generation and availability of molecular data, encompassing the analysis of diverse biomolecules such as DNA, RNA, proteins, and metabolites^1^. This has, consequently, enabled the study of fundamental processes such as gene expression^2^ and DNA methylation^3^, and opened new avenues for understanding complex biological systems and disease mechanisms. Profiling multiple ‘omics modalities in a disease cohort can provide a more comprehensive understanding of how distinct molecular processes operate in tandem to contribute to disease development and progression. Deriving such insights necessitates the development of methods for multimodal integration. Indeed, suitably designed integrative analysis can not only improve predictive outcomes but also help identify novel therapeutic targets, enabling the development of precision medicine^4^.

Integrating and analyzing multi-omics datasets poses significant computational challenges. These datasets are typically high-dimensional and heterogeneous, making dimensionality reduction and identification of shared patterns essential. Additionally, omics coverage may be incomplete, leading to missing data and potential biases. A variety of unsupervised methods have been proposed to address these challenges and derive insight from multi-omics datasets. The standard approach to dealing with the high-dimensionality of multi-omics data is to employ matrix factorization techniques. Methods such as MOFA (multi-omics factor analysis)^5^, iCluster^6^, and iNMF^7^ look for latent factors shared across data modalities. Another class of unsupervised methods attempts to produce a unified representation of heterogeneous ‘omics modalities by clustering samples based on similarities shared between their omics profiles—see, for instance, similarity network fusion (SNF)^8^ and unsupervised graph kernel learning approaches^9,10^.

In spite of their applications to a variety of bulk and single-cell multi-omics datasets for discovering molecular mechanisms and identifying biomarkers^11^, unsupervised methods do not allow one to detect signals or patterns pertinent to a specific target phenotype, such as a particular disease of interest. Meanwhile, methods for integrating heterogeneous data in the supervised setting are relatively sparse, where the challenge posed by the high-dimensionality of multi-omics data is further compounded by the small dataset size (i.e., the number of patient samples is significantly smaller than the total number of biological molecules profiled); particularly in the bulk ‘omics setting. Existing methods for supervised integration seek to exploit structures in ‘omics datasets: patients with similar ‘omics profiles are likely to share similar disease diagnoses. Based on this principle, several methods have been proposed to leverage graph neural networks (GNNs) to pose the task of patient phenotype prediction as a graph node classification problem. MOGONET^12^ leverages GNN feature extractors by using empirically generated patient similarity networks. MoGCN^13^, on the other hand, learns a unified GNN model using a patient similarity graph topology generated with SNF and low-dimensional node features learned through an autoencoder. While methods based on patient-similarity structures can alleviate the computational challenges associated with high-dimensionality in data features and low sample size, they do not leave any room for exploiting structures in the feature space, i.e., prior information about the relationship between biomolecules being measured.

In this work, we propose a novel explainable GNN framework, or GNNRAI (GNN-derived representation alignment and integration), for supervised integration of multi-omics data. Unlike existing methods, such as MOGONET and MoGCN, which use networks to model relationships among samples, we use graphs to model correlation structures among modality features (for example, genes in transcriptomics and proteins in proteomics data) from high-dimensional ‘omics data. These correlations among features reduce the effective dimensions of data. Combined with the message-passing mechanism in GNN, this enables us to analyze thousands of genes simultaneously using hundreds of samples in our analysis of AD multi-omics data. We leverage prior knowledge from recently published AD biological domains (biodomains or BDs)^14^, which are functional units in the transcriptome/proteome reflecting AD-associated endophenotypes. Each AD biodomain contains hundreds to thousands of genes/proteins, with co-expression relationships derived from existing protein-protein interaction databases used as graph topology. Given *k* ‘omics modalities, each sample is represented as *k* graphs in our framework. We leverage supervised GNNs to learn modality-specific low-dimensional graph embeddings. These low-dimensional embeddings are first aligned among data modalities to enforce shared patterns and then integrated using a set transformer^15^. The integrated multi-omics representations are used to predict the target phenotype. Our model architecture allows us to incorporate samples with incomplete ‘omics measurements and avoid a reduction in statistical power. We demonstrate the effectiveness of our framework by applying it to the task of predicting Alzheimer’s disease (AD) status by integrating transcriptomics and proteomics data from the Religious Order Study/Memory Aging project (ROSMAP) cohort. Proteomics data typically have a much smaller number of features relative to transcriptomics data, and this disparity is further exacerbated by the smaller number of samples with proteomic data in the ROSMAP cohort. Consequently, multi-omics integration methods might mask the role of proteomics modality^16^ if the useful information in transcriptomics and proteomics data is not aligned suitably. Our results show that proteomics data are more predictive than transcriptome data in the ROSMAP cohort. Our GNNRAI effectively integrates the heterogeneous data modalities, balancing the greater predictive power of proteomics over transcriptomics with larger information (sample size) available for transcriptomics, thereby improving prediction performance over the two unimodal models. Our modeling framework is compared to the MOGONET approach on held-out validation data and shows improved prediction metrics. To identify predictive modality features, we employ the method of integrated gradients^17^—a post hoc attribution method that utilizes gradients of the model prediction with respect to input features to estimate the relative importance of each feature (see ‘Methods’ for further details). Due to the incorporation of prior biological pathway knowledge in our GNNRAI, the identified biomarkers are predominantly functional, with nine well-known and eleven novel AD-related biomarkers among the top twenty. Finally, we probe our trained single-domain models to derive interactions between these domains using a set transformer in a second modeling stage. Interpretation of biodomain interactions via the method of integrated Hessians^18^ allows us to map how AD biodomains potentially interact to drive disease in combination.

## Results

### GNNRAI for supervised multi-omics integration and biomarker identification

In this work, we developed an AI framework, GNNRAI (GNN-derived representation alignment and integration), for performing supervised multi-omics data integration, accommodating potentially incomplete data, and identifying informative biomarkers and biological interactions. The backbone of our proposed method consisted of GNN-based feature extractor modules. Omics data, coupled with prior knowledge graphs, were processed through these GNN-based feature extractors to produce low-dimensional (sixteen for all the experiments) embeddings. Modeling the relationships between markers reduced the training sample size burden since correlation structure reduces the effective dimensions in high-dimensional omics data. Leveraging prior pathway knowledge and integrating multi-omics data maximized the likelihood that the identified informative features were functional.

**Fig. 1:**
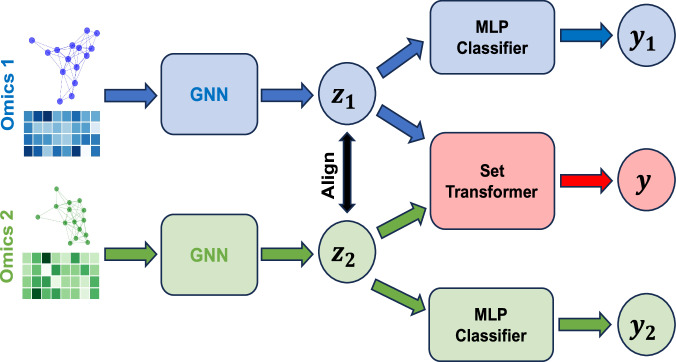
Schematic of our end-to-end integrative model GNNRAI. Data from individual ‘omics modalities are processed in their respective GNN feature extractors to produce low-dimensional embeddings (*z*_1_ and *z*_2_), which are then aligned and integrated through a set transformer. Samples with incomplete multi-omics measures have their embeddings processed through separate MLP (multi-layer perceptron) classifiers to produce modality-specific predictions of the target. Some elements of the figure were rendered using functions from Matplotlib in Python, while the entire figure was created in PowerPoint.

A schematic of our end-to-end GNNRAI model is shown in Fig. 1. This architecture facilitated efficient training on incomplete multi-omics datasets, as the feature extractor modules were updated by all samples regardless of the completeness of their omics data. Although we only demonstrated the integration of two modalities, it is feasible to extend this framework to accommodate additional modalities.

### Alzheimer’s disease patient classification datasets

In our study, we implemented the GNNRAI framework to integrate transcriptomic and proteomic data for the binary classification of Alzheimer’s disease within the ROSMAP cohort. We analyzed gene and protein data from the dorsolateral prefrontal cortex (DLPFC) brain region. The processing of the data and the criteria for AD diagnosis were elaborated in the ‘Methods’ section. The ratios of AD patients to healthy control were approximately 1.95:1 and 1.55:1 for transcriptomic and proteomic data, respectively. Building on Cary’s 2024 research on AD biodomains^14^, we created 16 datasets for AD classification, each containing measurements for the genes and proteins within a specific biodomain. All datasets contained the same set of participants and thus had the same class labels. The number of nodes (features) for the 16 BDs ranged from 45 to 2675 for transcriptomic and 41–1497 for proteomic data, respectively. Refer to the Methods section for details on AD biodomains and Table 3 for graph sizes (number of nodes and edges) for each biodomain. After data processing, we had 228 samples with both transcriptomic and proteomic data, 59 with only proteomic data, and 336 with only transcriptomic data. Each sample’s omics data was coded as a set of graphs, one for each available modality per biodomain. In those graphs, nodes represented genes or proteins from that biodomain, with their expression or abundance encoded as a node feature. These graphs were structured by the biodomain’s knowledge graph from querying the Pathway Commons database^19^. Each sample was labeled with a binary indicator to denote whether it was from an AD patient or a healthy control.

### The proposed GNNRAI AI framework outperformed the benchmark MOGONET method on AD/control classification

**Fig. 2:**
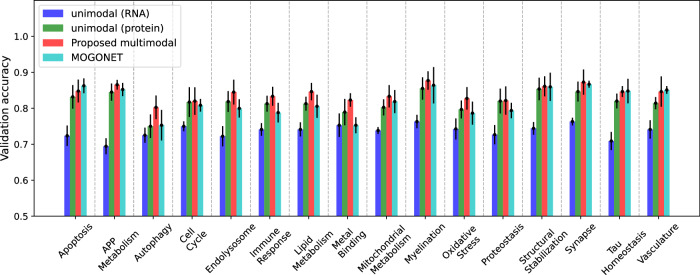
Three-fold cross-validation performance of our proposed integrative model (red) compared to that of the benchmark MOGONET model (cyan) on the set of common samples having both proteomics and transcriptomics measurements. The performance of the integrative models is also compared to unimodal GNN transcriptomics (blue) and proteomics (green) models.

The *multi-omics graph convolutional network*, or MOGONET^12^, was a supervised learning framework for integrating multi-omics data using GNNs. MOGONET processed individual modalities separately by constructing patient similarity networks using the cosine distance metric to assign edges. Graph neural networks operated on these patient similarity networks to make modality-specific predictions, which were then integrated through a view correlation discovery network (VCDN). In contrast to MOGONET, our approach imposed a network topology over the space of input features within each modality. The MOGONET architecture made it implausible to incorporate priors on the space of features (such as AD biodomains). Furthermore, MOGONET required samples to have complete measurements (i.e., no missing modalities). We trained our unimodal and integrative models on the set of samples with both transcriptomics and proteomics measurements for each of the 16 BDs and compared their validation predictive performance to that of MOGONET trained on the same datasets. A comparison of the threefold cross-validation prediction accuracy between these models is shown in Fig. 2 and Supplementary Table 3. On average, across 16 BDs, our integrative GNNRAI increased validation accuracy by 2.2% over MOGONET. We observed that when trained on an equal number of samples, unimodal proteomics models consistently outperformed unimodal transcriptomics models with an average increase in validation accuracy of 8.2%. Despite having fewer features (see Table 3), proteomics data were more predictive in the ROSMAP cohort, aligning with^20^. Our integrative models outperformed the integrative MOGONET models in 13 out of 16 BD datasets (except for *apoptosis, Tau homeostasis,* and *vasculature)* by an average of 2.9%. Additionally, for seven BD datasets (*cell cycle*, *endolysosome*, *immune response*, *lipid metabolism*, *metal binding*, *oxidative stress,* and *proteostasis*), our unimodal proteomics models surpassed the multimodal MOGONET models by 1.9% on average. This was likely because proteomics and transcriptomics data were not always consistent, and MOGONET integrated modality-specific predictions rather than modality representations. In contrast, our framework’s integration of representations of transcriptomics and proteomics modalities improved the unimodal predictive performance across all 16 BD datasets, demonstrating effective integration.

### Multi-omics GNNRAI models outperformed unimodal GNNRAI models trained on transcriptomics and proteomics alone

For samples with complete measurements, our model’s performance was evaluated using the held-out validation set predictions from the integrative component (i.e., the set transformer in Fig. 1). For samples with incomplete measurements, predictions were based on their respective unimodal classifiers. We found that integrating the two modalities resulted in better-performing classifiers compared to the unimodal counterparts. This finding was significant given that we had 564 samples with transcriptomic data but only 287 with proteomic data. A larger number of less predictive transcriptomic samples could obscure the superior performance of proteomic samples if the useful information from both modalities was not effectively aligned and integrated.

**Fig. 3:**
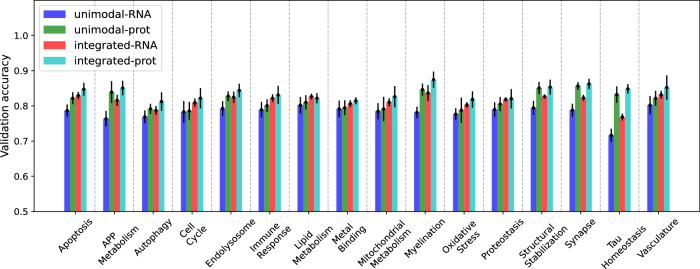
Three-fold cross-validation performance of the integrative, multimodal model (red and cyan) compared to that of unimodal GNN models (blue and green) trained on the incomplete multi-omics datasets for 16 AD biodomains. The performance of the multimodal model is calculated on two sets of validation samples – the set of all validation samples with transcriptomics and that with proteomics measurements.

To ensure a fair comparison, we used two sets of validation samples for evaluating the performance. The first validation dataset comprised samples with transcriptomics measurements (used to test the unimodal transcriptomics GNN models). The second validation dataset included samples with proteomics measurements (used to test the unimodal proteomics models). Figure 3 and Supplementary Table 4 compare the validation accuracy of GNN models trained solely on transcriptomics and proteomics samples with the end-to-end multimodal models trained on all samples. Validation performance for unimodal models was denoted as ‘unimodal-RNA’ and ‘unimodal-prot’, while the two validation scores from the integrative model were denoted as ‘integrated-RNA’ and ‘integrated-prot’, respectively. In spite of the smaller training dataset, ‘unimodal-prot’ consistently outperformed ‘unimodal-RNA’ across all 16 BDs by an average of 3.4%, reiterating that proteomics data provided more AD-predictive information than transcriptome data in the ROSMAP cohort. When we compared the multimodal to unimodal performance, ‘integrated-RNA’ consistently surpassed ‘unimodal-RNA’ across all 16 BDs by an average of 3.3%, demonstrating that integrating proteomics with transcriptomics data enhanced classification performance. Similarly, there was a consistent performance improvement from ‘unimodal-prot’ to ‘integrated-prot’, an average of 2.1%, albeit less pronounced than in the RNA modality. Furthermore, ‘integrated-prot’ was generally better than ‘integrated-RNA’ by an average of 2.2%. The only exception was for the *lipid metabolism* BD, despite the fact that the proteome-specific classifier was trained on only 59 samples compared to 336 samples for the transcriptome-specific classifier. This suggests that the target-predictive signals from transcriptomic and proteomic embeddings were aligned and integrated effectively, resulting in smaller performance differences between samples with transcriptomic data and proteomic data than in unimodal transcriptomic models.

### Validation on ROSMAP, Mount Sinai Brain Bank (MSBB), and Mayo Clinic transcriptomics and proteomics data

To validate the predictive ability of our models trained on ROSMAP DLPFC samples, we curated samples with transcriptomics and/or proteomics measurements from the following studies and brain regions -

1. ROSMAP samples from the *anterior cingulate cortex* (ACC) and *posterior cingulate cortex* (PCC) brain regions with transcriptomic measurements.
2. MSBB^21^ samples from the *parahippocampal gyrus* (PHG), *frontal pole* (FP), *inferior frontal gyrus* (IFG), and *superior temporal gyrus* (STG) brain regions. Only PHG tissue had both transcriptomic and proteomic data, while the remaining tissues had transcriptomics only.
3. Mayo Clinic^22^ samples from the *temporal cortex* (TCX) brain region. Although TCX tissue had both transcriptomic and proteomic data, the proteomics measurements were acquired by label-free quantification, different from the tandem mass tag (TMT) quantification platform used in ROSMAP and MSBB. Hence, we did not validate ROSMAP-derived models on Mayo proteomics data.

Table 1 shows the sample counts of the curated validation datasets. The procedure for annotating MSBB and Mayo samples with ground truth labels were described in the Methods section. We noted that MSBB and ROSMAP adopted similar diagnostic criteria, while Mayo clinic cohort followed Mayo neurologist guidelines^23^.

**Table 1:** Transcriptomics and proteomics sample count in validation data

Tissue	ROSMAP (ACC)	ROSMAP (PCC)	MSBB (PHG)	MSBB (FP)	MSBB (IFG)	MSBB (STG)	Mayo (TCX)
RNA	354	332	122	106	106	108	62
Protein	–	–	122	–	–	–	

**Fig. 4:**
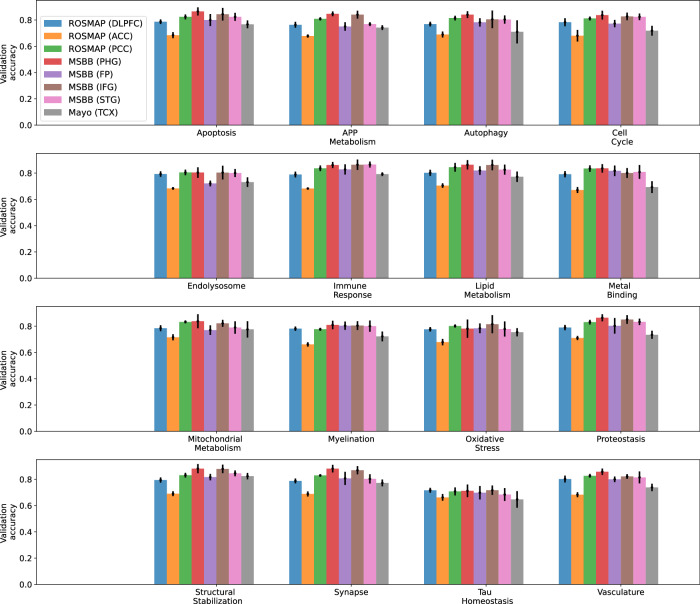
Predictive performance of applying unimodal transcriptomics model trained on ROSMAP DLPFC training dataset to validate transcriptomics samples from various cohorts and brain tissues.

We first applied the ROSMAP DLPFC-trained transcriptomics model to transcriptomics data from ROSMAP ACC and PCC brain regions, MSBB PHG, FP, IFG, and STG brain regions, and Mayo TCX brain regions. For comparison, we also included the predictive accuracy of the ROSMAP DLPFC validation dataset (Fig. 4, Supplementary Table 5). Figure 4 shows that the same GNN model has different predictive performances on different brain regions. Generally, ROSMAP PCC had slightly higher predictive accuracy (cross-BD average of 0.81) than DLPFC (0.78), which was, in turn, higher than the predictive accuracy of ACC (0.69). For MSBB, PHG had the highest predictive accuracy (0.84), followed by IFG (0.83) and STG (0.80), which predicted better than FP (0.79). MSBB PHG had the highest predictive accuracy across the three cohorts. MSBB IFG and STG had a comparable performance with ROSMAP PCC, while MSBB FP had a comparable performance with ROSMAP DLPFC, which predicted better than Mayo TCX (0.74). ROSMAP ACC had the lowest predictive accuracy on average. Nevertheless, ROSMAP ACC predictive accuracy was better than a random guess. The different predictive performance on transcriptomic data from different brain regions might be explained in terms of neuropathological burdens – the FP and DLPFC regions are impacted at a similar disease stage, whereas the PHG tends to be affected much earlier in disease progression. This could be further exacerbated by the differences in cohort sample selection between ROSMAP and MSBB. MSBB samples were selected for multi-omics profiling based on the presence of remarkable AD neuropathology, whereas the ROSMAP study was a longitudinal cohort and did not pre-select samples for extreme neuropathology. Thus, disease signatures learned in the ROSMAP DLPFC samples could be amplified in the MSBB samples, leading to an increase in the predictive accuracy of our models. This also implied that transcriptomic signatures might be translational across relevant brain tissues.

**Fig. 5:**
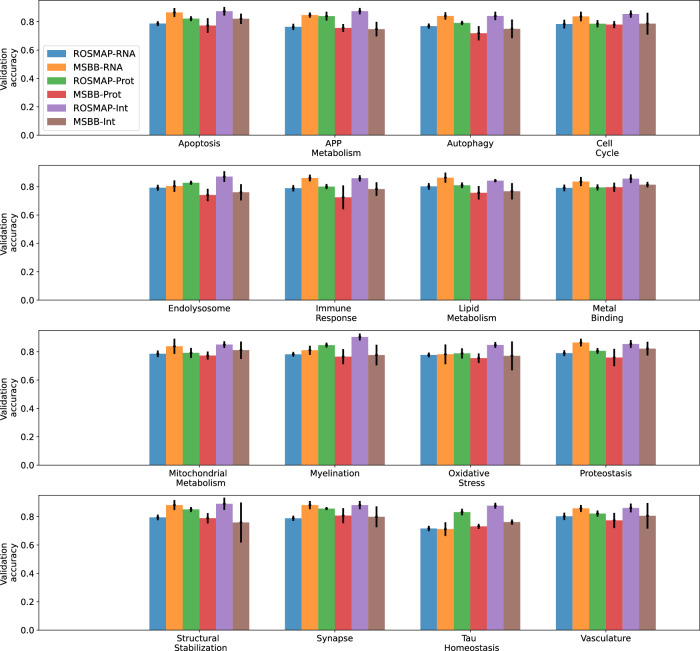
Predictive performance of applying unimodal and multimodal models trained on ROSMAP DLPFC training dataset to samples from ROSMAP DLPFC validation and MSBB PHG validation datasets. Prot protein, Int integrative.

Next, we applied the ROSMAP DLPFC-trained proteomics and integrative models to proteomics and multi-omics data from the MSBB PHG brain region, respectively (Fig. 5, Supplementary Table 6). We also included ROSMAP DLPFC and MSBB PHG transcriptomics performance in Fig. 5 for comparison. MSBB PHG had lower predictive accuracy than ROSMAP DLPFC for proteomics and integrative models by an average of 5.4% and 8.1%, respectively. Unlike in ROSMAP, where proteomics data had higher predictive accuracy than transcriptomics data, and the integrative model improved upon the two unimodal models, proteomics data were less predictive than transcriptomics data by an average of 7.4%, and the integrative model had lower predictive accuracy than the proteomics model for 9 AD biodomains in MSBB. This might be due to the fact that the GNN models were trained on a much smaller set of ROSMAP proteomics data (two-thirds of total samples: 287 × 2/3 = 191) than ROSMAP transcriptomics data (two-thirds of total samples: 564 × 2/3 = 376), causing poor generalization performance on unseen data.

### Identification of biomarkers relevant to AD

We applied the integrated gradients method to our trained multi-omics GNNRAI models to derive importance scores on input graph nodes (genes and proteins). We used a permutation-based approach to determine the importance score threshold by controlling the false discovery rate (FDR) to be below 0.05. Like standard permutation procedures for multiple hypothesis testing, we treated the original importance scores as the observed test statistics, generated 300 permuted datasets by randomly permuting the training labels, and trained our integrative models on the permuted datasets. The resulting models were called null models. The importance scores for all genes/proteins in a graph from each sample of the 300 null models were used as background test statistics. Since we calculated FDR rather than corrected *p*-values, the estimated empirical FDR was confidently accurate for *B* ≥ 100^24^. We took *B* = 300 in all our experiments. See ‘Methods’ for the procedure to calculate permutation-based FDR.

We determined whether a gene/protein was informative only in correctly predicted validation samples. The top 20 AD-predictive genes/proteins are shown in Table 2. To rank the informative genes/proteins identified across the analyses, we added the number of unique sample identifiers for which a given gene/protein was identified as informative across modalities and 16 AD biodomains for each study (columns of Total samples in Table 2), divided by the number of correctly predicted validation samples in each study to obtain the fraction of samples for each gene/protein where it is informative, then calculated the average fraction across studies, based on which genes/proteins were ranked.

**Table 2:** Top 20 informative genes across studies

Rank	Gene/ proteins	Average fraction of samples	# ROSMAP DLPFC samples	# MSBB PHG samples
1	MDK	0.84	118	65
2	APP	0.672	109	43
3	NTN1	0.62	92	45
4	GFAP	0.569	67	52
5	ICAM1	0.541	66	48
6	LTF	0.532	67	46
7	FLNA	0.466	33	56
8	CD44	0.46	43	49
9	APOE	0.459	54	42
10	IL1B	0.42	76	22
11	VGF	0.415	52	36
12	FLT1	0.39	68	22
13	PTN	0.382	48	33
14	IQGAP3	0.364	58	24
15	LGMN	0.35	59	21
16	GJA1	0.347	34	36
17	PRKCD	0.344	33	36
18	GIPR	0.34	58	20
19	TAC1	0.336	23	41
20	ADCYAP1	0.335	34	34

The top-ranked gene in these analyses was *MDK*, which was informative in the binary classification task for 183 unique validation samples (an average of 84% of correctly predicted validation samples). *MDK* is a secreted growth factor that has consistently been identified among a suite of matrisome proteins that associate with Aβ plaques (i.e., Module M42 in refs. ^20,25^), and has been shown to influence the aggregation of amyloid-beta, both in vitro and in vivo^26^. Notably, there are also five other proteins from the M42 module among the top 20 genes (APP, NTN1, APOE, FLT1, and PTN), and module M42 proteins are significantly enriched among the most informative genes (GSEA adjusted p-value = 0.004). *VGF* was another top-ranked gene in these analyses and has consistently been identified as a robust biomarker for AD^27,28^, as well as being a top predicted regulator of multiscale AD networks^29^.

The four genes in the top 20 that had the highest integrated AD Target Risk Score (TRS^14^), were *APP*, *APOE*, *LGMN*, and *LTF*. Each of these genes was informative for over 80 total samples and had TRS in the top 2% of all scored genes. *APP* is a well-known disease gene that is the proteolytic precursor of the Aβ peptide, which is a major component of one of the hallmark neuropathologies of the disease, and variants within *APP* are causal for rare autosomal dominantly inherited forms of AD. Distinct alleles of the *APOE* gene represent both the strongest genetic risk and strongest genetic protective factors in genome-wide association studies of late-onset AD^30^, and other APOE alleles have been found to be suppressors of mutations causing autosomal dominant AD^31^. *APOE* has been implicated in several disease endophenotypes, including the accumulation of both Aβ plaques and tau neurofibrillary tangles, mediating glial cell response, and disturbances to the blood-brain barrier that occur during AD pathogenesis^32^. *LTF*, or lactotransferrin, has recently been identified as a predictor of Aβ burden^33^. *LGMN*, also known as δ-secretase, is an asparagine endopeptidase that is involved in the cleavage of both tau^34^ and *APP*^35^ proteolysis, which is linked to increased pathogenicity in each case. This corresponded with the findings from the individual gene/protein analyses where *LGMN* was the most impactful in Tau Homeostasis and APP Metabolism biodomains.

The remaining eleven genes from the top 20 were novel candidate biomarkers that have not been previously linked to AD pathogenesis in published studies. However, their predictive potential for AD suggested they warrant further investigation. For example, *IQGAP3* was ranked #14 in this analysis, had a TRS in the top 10%, and was differentially expressed in both transcriptomics and proteomic samples. Despite having no publications where *IQGAP3* is implicated in AD, it was linked with cytoskeletal maintenance and neurite outgrowth^36^, which is consistent with its role in the Structural Stabilization domain in these analyses.

At least nine genes among the top 20 were strongly related to AD biology. Moreover, among the six M42 module members that were ranked top 20, three (APOE, FLT1, PTN), unlike MDK, NTN1, and APP, did not show high magnitude fold changes when comparing AD patients with healthy controls (ranging from 1.11 to 1.27 in proteomic data). These demonstrated that our integrative method successfully identified functional features of small effects by incorporating prior biological pathway knowledge.

### Detecting interactions among biodomains

For a multi-modal integrative model trained on a given biodomain, the class token representation from the final set transformer was a single low-dimensional embedding that unified information across modalities within the biodomain. We, therefore, collected class token representations in integrative models from all 16 BDs and trained an auxiliary set transformer to integrate information across biodomains. Integrated Hessians^18^ was applied to this second set transformer model to derive interaction scores between its input tokens. The biodomains partition gene functions into distinct molecular endophenotypes. However, these endophenotypes can and do interact during the etiology of the disease. Therefore, a primary goal of utilizing the biodomain framework is to identify interactions between domains that could broaden our understanding of disease development.

**Fig. 6:**
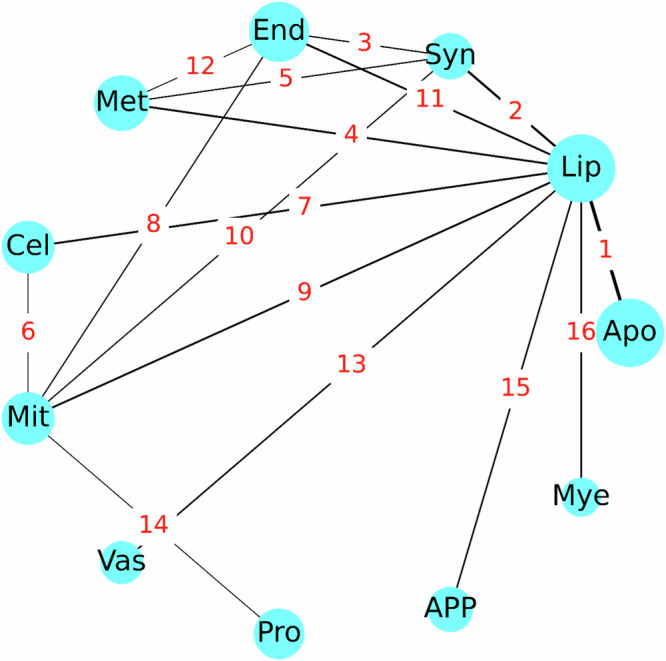
Interactions between biodomains identified by Integrated Hessians analysis on a multi-modal model trained on samples with complete multi-omics. The nodes represent biodomains, and the edges represent interactions, with the edge annotations being the rank of the interaction. The biodomain names based on the 3-letter abbreviations are: apoptosis (apo), lipid metabolism (lip), synapse (syn), endolysosome (end), metal binding (met), cell cycle (cel), mitochondrial metabolism (mit), myelination (mye), vasculature (vas), proteostasis (pro) and APP metabolism (app). The figure was rendered using functions from Matplotlib in Python.

The top interactions detected are shown as a graph where each node is a BD in Fig. 6. Interactions were ranked by repeating the model training and informative interaction identification process ten times with different random weight initializations. From each iteration, the top ten percent of interactions, determined by ranking the number of samples for which each interaction was informative, were noted. Interactions present in the top ten percent three or more times out of ten were recorded. Rank was determined first by the number of appearances in the top ten percent, then, in the case of ties, by the total number of samples in which the interaction was informative. The domain nodes with the largest degree were Lipid Metabolism (degree = 9), followed by Mitochondrial Metabolism (degree = 5), Synapse, and Endolysosome (degree = 4, each). The centrality of these domains was supported by the observation that Synapse, Lipid Metabolism, and Mitochondrial Metabolism were among the top risk-enriched biodomains^14^. The observation that Lipid Metabolism was a hub in this graph suggested that aspects of Lipid Metabolism influenced many other disease processes. The centrality of Lipid Metabolism to AD pathogenesis was supported by myriad observations from recent decades, including genetic studies that implicate Lipid Metabolism associated genes (e.g., *APOE*, *CLU*, *ABCA7*, *SORL1*) in driving AD risk^37^, the observation that amyloid-beta production occurs in lipid raft membrane microdomains^38^, recent lipidomic studies that identify changes in lipid species that are specific to the disease^39,40^, and many more.

Other interaction relationships represented in this graph were informative and supported by evidence from the literature. As mentioned above, the edge between APP Metabolism and Lipid Metabolism was supported by the influence of lipid rafts on amyloid-beta production. The link between Lipid Metabolism and Mitochondrial Metabolism was also very well supported given that β-oxidation of fatty acids, which is the primary catabolic pathway, occurs in the mitochondrial matrix. Given that mitochondria provide the requisite energy and precursor metabolites for cell cycle progression, and that mitochondrial biogenesis and dynamics are influenced by cell cycle regulators, the interdependence of Cell Cycle and Mitochondrial Metabolism was also well supported. The top-ranked edge between Lipid Metabolism and Apoptosis evoked ferroptosis, which is an iron-dependent cell death mechanism that is distinct from apoptosis but involves the accumulation of peroxidated lipid species^41^ and is the focus of newly emerging hypotheses of disease pathogenesis^42^. Further, supporting this association is the observation that 6 of the top 20 identified informative genes – i.e., *LGMN*^43,44^, *LTF*^45,46^, *CD44*^47,48^, *PRKCD*^49^, *IL1B*^50,51^, and *GJA1*^52–54^—are associated with regulating aspects of ferroptosis in diverse contexts. It was noted that the immune response biodomain, which is strongly involved in AD, is absent in Fig. 6. The interplay between the immune response and lipid metabolism BDs ranked seventeen in these analyses—the highest-ranking interaction not included in Fig. 6. The reason for this omission in our list of top pairwise interactions remains unclear and warrants further investigation which is beyond the scope of our current work.

## Discussion

In this work, we proposed an end-to-end AI framework, or GNNRAI, for supervised alignment and integration of multi-omics data with prior biological information expressed as knowledge graphs. Our method was based on the use of GNNs for learning low-dimensional embeddings from high-dimensional ‘omics data and could accommodate samples with missing modalities. Using the ROSMAP data, we curated 16 binary classification datasets – each dataset comprising a view of the ROSMAP gene expression and protein abundance data within an AD-associated biodomain. We noted that the size of biological domains varied significantly from smallest to largest domains and therefore allowed us to test the robustness of our approach to varying input dimensionality. Our approach has validated its efficacy in the task of integrating transcriptomics and proteomics data from the ROSMAP cohort. It has outshined the benchmark MOGONET method in 13 of the 16 BDs and shown improvements over the two unimodal models for all 16 BDs. This outcome is noteworthy considering the disparity in sample sizes, with 564 transcriptomic samples and only 287 proteomic samples. The abundance of less predictive transcriptomic samples could potentially conceal the enhanced performance of the proteomic samples unless the valuable insights from both data types are properly synchronized.

The task of integrating multi-omics data is computationally challenging for several reasons. We demonstrated that one of the challenges, the curse of dimensionality arising from the large number of ‘omics features relative to the sample size, could be overcome by leveraging the correlation structure in graphs and message passing in GNNs. Unlike unsupervised learning, the 16-dimensional embeddings formed under the supervision of disease status (model target), are expected to be related to disease status. As an illustration, we calculated the principal components (PC) of the embeddings for small biodomain APP metabolism, medium-sized biodomains endolysosome and immune response, and large biodomain, synapse. The plots of PC1 vs. PC2 for training and validation data are shown in Supplementary Fig. 1. Indeed, AD patients and healthy controls clustered separately, although not perfectly, in the reduced PC space. Additionally, we showed that the separation of feature extraction modules and set transformer-based integration allowed us to utilize samples with missing modalities—a characteristic feature of multi-omics datasets.

Our framework allows one to integrate modalities where prior information about the relationship between input features can be expressed in the form of knowledge graphs. We leveraged existing work on AD biodomains to extract network topologies for transcriptomics and proteomics modalities. Our approach did not make a distinction in the structure of the knowledge graphs used for these modalities, thereby implicitly imposing a simplified assumption that network relationships between transcripts are exactly reproduced in the proteins they code for. Furthermore, we did not incorporate data from other modalities within the ROSMAP study, such as methylomics and metabolomics, due to the current unavailability of direct prior knowledge graphs for these modalities. However, existing transcriptomic and proteomic networks can be leveraged for the construction of gene-centered methylomics and metabolomic knowledge graphs. For instance, metabolites catalyzed by the same genes/proteins may be determined to share a relationship. For genes that have an edge in a transcriptomic network, CpG sites within their regulatory regions (promoters, enhancers, etc.) may be determined to share the same edge within the corresponding methylation network. On the other hand, the number of genetic variants (including copy number variations) is much larger than that of proteins or genes, and most of them are in non-coding regions. Considering all genetic variants in our graphs is a challenge. One potential approach to accommodate genetic variants is to consider cis-genetic variants that were identified to affect gene expression levels or protein abundance. This way, we can still construct gene-centered prior knowledge graphs. Finally, we identified informative features through the model-agnostic method of integrated gradients which derived importance scores on individual graph nodes independently. Interpretation of GNN predictions can, in theory, be enhanced by using an explanation method to identify informative subgraphs or *motifs*. While methods in this direction did exist^55^, our experience was that we were unable to extract meaningful subgraphs/motifs through the application of such methods. How to identify correlated informative features efficiently is one of the important future research directions for GNNs.

## Methods

### Data preprocessing

To investigate AD mechanisms, we adopted a combination of clinical and neuropathological criteria used in ref. ^20^ to assign ground truth labels (AD case or control) to patients within the ROSMAP cohort. In particular, we used clinical cognitive tests, such as MMSE (the Mini-Mental State Examination^56^) or CDR (Clinical Dementia Rating) to assess dementia: MMSE score ≤ 24 or CDR ≥ 1. Neuropathological assessment of patients was conducted post-mortem using Braak staging^57^ and CERAD (The Consortium to Establish a Registry for Alzheimer’s Disease) scoring^58^ to reflect AD hallmarks. CERAD scores 0–3 correspond to no AD/none, possible/sparse, probable AD/moderate, and definite/frequent, respectively. The Braak score, or seven Braak stagings, classifies the severity and distribution of tau pathology in the brain. Cases with CERAD 0–1 and Braak 0–3 without dementia at the last evaluation were annotated as controls (if Braak score equals 3, then CERAD must equal 0); cases with CERAD 2–3 and Braak 3–6 with dementia at last evaluation were annotated as AD.

Downloaded RNA-Seq count data were log2 transformed and corrected for age, sex, and postmortem interval (PMI) covariates. Downloaded protein abundance data were log2 transformed and median zero centered per feature. Finally, age, sex and postmortem interval (PMI) were regressed out.

For validation of MSBB samples, we used similar diagnostic criteria, except MMSE was replaced with CDR^21^ since MSBB did not provide MMSE information. MSBB gene expression/protein abundance data were processed similarly to ROSMAP data. In addition, the patient’s race was regressed.

For patients in the Mayo study, AD and controls were taken to be the reported diagnosis according to Mayo neurologist guidelines, as described in ref. ^23^. In contrast to ROSMAP and MSBB, which made use of tandem mass tag (TMT) quantification, Mayo proteomics data were acquired with label-free quantification, hence we did not validate our models on Mayo proteomics data. Mayo gene expression data were processed similarly to ROSMAP data.

Access to the multi-omics data for the three cohorts was approved under local IRB Number 2022-DAR-053 AMP-AD.

### Network priors from Alzheimer’s disease biological domains

The prior biological knowledge ascribed to nodes and edges in the knowledge graphs used for the analysis was derived from publicly accessible biological databases. These graphs provided a topological organization to the biological domains (or biodomains), which were 19 AD-associated endophenotypic descriptors, such as immune response and mitochondrial metabolism^14^. The biodomains were lists of functional biological definitions describing aspects of AD and were defined with suites of relevant Gene Ontology (GO) terms^59^. Each GO term was annotated with a set of genes, and biological processes within a domain that was enriched for composite metrics of disease risk^14^ could be identified using standard enrichment procedures. We used significantly enriched GO terms (gene set enrichment analysis adjusted p-value < 0.01 and normalized enrichment score > 1.7) – 16 of the 19 biodomains had GO terms that met these criteria – and extracted the leading edge genes from each term to seed knowledge graph generation through a pathway reconstruction pipeline. We performed the shortest path reconstruction among all risk-enriched genes for each domain using protein-protein interaction (PPI) edge annotations from the Pathway Commons database^19^, version 13. Given the nonlinear relationships implicated in most biological interactions, the shortest path to connect two genes was selected for creating an edge between two nodes. For a given protein, expressed by a gene in the biodomain, an edge was derived from the larger PPI network. The final network object consisted of edges, which were the PPI, and nodes, which were the GO term-derived gene list. See the ‘Data Availability’ section for the website containing the network files for the 16 biodomains. A summary of the sizes of the transcriptomics and proteomics prior networks within each biodomain is shown in Table 3.

**Table 3:** Summary of AD biodomain knowledge graphs

Biological domain	Nodes (mRNA)	Edges (mRNA)	Nodes (Proteins)	Edges (Proteins)
APP Metabolism	147	316	87	154
Apoptosis	958	10,963	451	3261
Autophagy	505	2665	304	1096
Cell Cycle	728	6561	337	1779
Endolysosome	1096	8909	665	3874
Immune Response	1507	16,354	688	4683
Lipid Metabolism	1671	13,515	891	4366
Metal Binding and Homeostasis	801	4822	441	1398
Mitochondrial Metabolism	1273	7092	858	3493
Myelination	189	348	120	132
Oxidative Stress	346	2080	185	637
Proteostasis	2675	29,812	1477	13,323
Structural Stabilization	2245	23,850	1297	12,163
Synapse	2067	21,056	1234	9686
Tau Homeostasis	45	90	41	81
Vasculature	756	7098	356	2008

### Modeling framework for multi-omics integration

In this work, we proposed an end-to-end framework for supervised integration of incomplete multi-omics data. Our modeling framework comprised two key components: (1) graph neural network-based feature extractors and (2) feature alignment as well as set transformer-based feature integration among modalities.

#### Graph Neural Network-based feature extractors

**Fig. 7:**
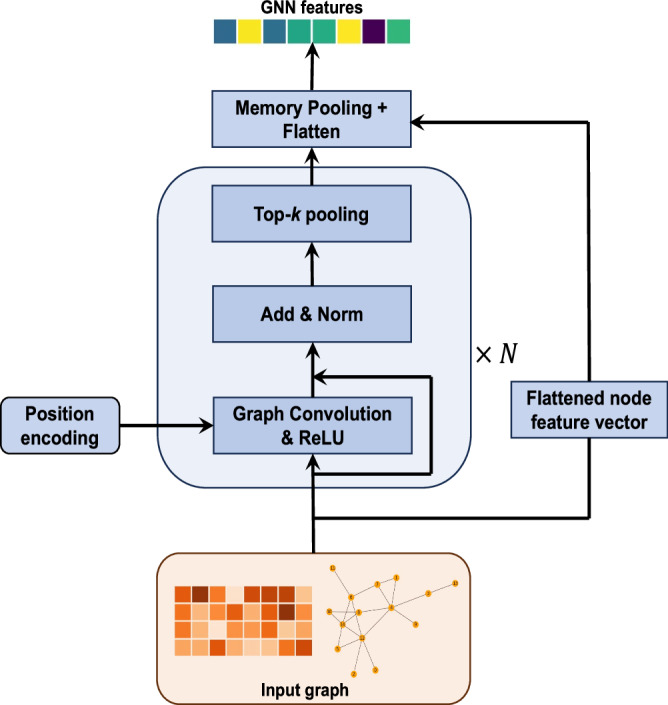
Schematic of GNN-based feature extractor. The feature extractor is comprised of a stack of graph convolution blocks, a memory pooling layer, and a residual connection between the original input node features and the output of the memory pooling layer. Some elements of the figure were rendered using functions from Matplotlib in Python, while the entire figure was created in PowerPoint.

Let ${{\boldsymbol{x}}}_{i}\in {{\mathbb{R}}}^{{d}_{i}}$ be the set of features for the ${i}^{{th}}$ modality, and ${\mathcal{G}}$ be an undirected graph with ${d}_{i}$ nodes, with each node representing a feature in the ${i}^{{th}}$ modality. ${\mathcal{G}}$ may be constructed empirically via binarizing the matrix of correlation coefficients among features or may represent prior knowledge on the space of features defining known pairwise relationships. Let ${\mathcal{E}}$ be the list of edges in ${\mathcal{G}}$ and ${\mathcal{W}}$ be an optional list of corresponding vector-valued edge weights such that $\left|{\mathcal{W}}\right|=\left|{\mathcal{E}}\right|$. Given a sample of ‘omics measurements ${{\boldsymbol{x}}}_{i}$ and an associated graph topology ${\mathcal{G}}$, we set up a graph neural network (GNN) and learned ${{\boldsymbol{z}}}_{i}=g\left({{\boldsymbol{x}}}_{i},{\mathcal{E}},{\mathcal{W}}\right)$, where ${{\boldsymbol{z}}}_{{\boldsymbol{i}}}\in {{\mathbb{R}}}^{m}$ is a GNN-derived embedding with dimension $m$. A schematic of the GNN-based feature extractor is shown in Fig. 7.

First, we constructed a positional encoding of ${d}_{i}$ features into $c$ ($c\, < \,{d}_{i}$) learnable communities using a standard lookup table embedding function in *Pytorch*, which was softmax-normalized. The input node feature vector ${{\boldsymbol{x}}}_{i}$ to $g$ was linearly transformed by using the $k$ communities as bases, followed by passing through a stack of graph convolution blocks. Each block (round-corner rectangular box in Fig. 7) comprised three sub-blocks. The first sub-block was a message passing graph convolution layer, followed by a ReLU activation. The second sub-block was a residual connection adding input node features to the output of ReLU, followed by batch normalization. The third sub-block was an optional top-*k* pooling layer^60^, which we applied to mask non-informative nodes when input graphs were large. Specifically, the parameters in the top-*k* pooling layers were set so that the graphs before the memory pooling layer contained at most 300 non-masked nodes. Input node features, which passed through $N$ graph convolution blocks, were transformed into latent node features ${\widetilde{{\boldsymbol{x}}}}_{i}\in {{\mathbb{R}}}^{{d}_{i}}$. The associated graph topology remained unchanged since we did not employ edge-updating in our model. The transformed node feature vector ${\widetilde{{\boldsymbol{x}}}}_{i}$ passed through a memory pooling layer^61^ which learned a coarse graph representation through soft cluster assignments. Subsequently, ${\widetilde{{\boldsymbol{x}}}}_{i}$ was reduced to ${{{\boldsymbol{z}}}^{{\boldsymbol{{\prime} }}}}_{i}\in {{\rm{{\mathbb{R}}}}}^{{d}_{i}{\prime} \times {d}_{c}}$, where ${d}_{c}$ is the number of clusters, and ${d}_{{i}^{{\prime} }}$ is the number of features in a cluster, which was then flattened. Lastly, a residual connection was employed, adding the linearly transformed original input node feature vector to the flattened ${\widetilde{{\boldsymbol{z}}}}_{i}$, which was subsequently linearly mapped to ${{\boldsymbol{z}}}_{i}\in {{\mathbb{R}}}^{m}$.

#### Graph convolution layer

In a standard graph convolution layer, the features of each node are first linearly transformed by the same weights, followed by a message-passing update. To enhance the expressive capacity of the GNN, we softly mapped nodes into (*c*) communities, each with its own learnable weights for linearly transforming node features^62^. The transformation of a node’s features depended on its membership coefficients belonging to communities. In particular, let ${h}_{{in}}\in {{\mathbb{R}}}^{R\times {C}_{{in}}}$ denote the input matrix to our graph convolution layer, where $R$ was the number of nodes, and ${C}_{{in}}$ represented the number of features in an input node. Let ${C}_{{out}}$ be the number of features in an output node of a graph convolution layer. Suppose $R$ nodes were mapped to $c$ communities, and the softmax-normalized positional encoding was $p\in {{\mathbb{R}}}^{R\times c}$. We first mapped the input node features linearly: ${h}^{{\prime} }={h}_{in}{W}_{c}$, where ${W}_{c}\in {{\mathbb{R}}}^{{C}_{{in}}\times {\widetilde{C}}_{{out}}}$ were learnable weights, ${\hat{C}}_{{out}}=c\,\cdot \,{C}_{{out}}$, ${h}^{{\prime} }\in {{\rm{{\mathbb{R}}}}}^{R\times {\tilde{C}}_{out}}.$ Linearly transformed node features $h{\rm{{\prime} }}$ were then reshaped to ${h}^{{\prime} }\in {{\rm{{\mathbb{R}}}}}^{R\times c\times {C}_{{out}}}$. The positional encoding was then used to linearly weight node features along each output channel - ${\hat{h}}_{:,i}=\sum {{h}^{{\prime} }}_{:,:,i}\odot {unsqueeze}(p)$, where $\odot$ was the element-wise product operation, *unsqueeze()* was a Pytorch function that added a dimension to a tensor so the dimensions of operators of product operations were compatible, and the summation was among $c$ communities. The resulting node features were $\hat{h}\in {{\mathbb{R}}}^{R\times {C}_{{out}}}$. Finally, a message-passing update on the node features was performed:

1 $${h}_{{out},j}=b+\sum _{r\in \{j\}\cup {{\mathscr{A}}}_{j}}\frac{{e}_{r,j}\,\cdot \,{\hat{h}}_{j}}{{\hat{d}}_{r}{\hat{d}}_{j}},$$

where ${\hat{h}}_{j}\in {{\mathbb{R}}}^{{{\rm{C}}}_{{\rm{out}}}}$ was the feature vector of the ${j}^{{th}}$ node, ${{\mathscr{A}}}_{j}$ was the set of indices of the neighbor nodes to node $j$, ${\hat{d}}_{j}$ and ${\hat{d}}_{r}$ were the degrees of nodes $j$ and $r$ respectively, ${e}_{r,j}{\mathbb{\in }}{\mathbb{R}}$ was the attribute of the edge connecting nodes $j$ and $r$, and $b\in {{\mathbb{R}}}^{{{\rm{C}}}_{{\rm{out}}}}$ was a learnable intercept. Note that our graph convolution layer was an extension of the standard graph convolution of ref. ^63^, where we allowed for the inclusion of positional encoding.

#### Aligning embeddings among modalities

Data modalities may be heterogeneous, and each dimension of the GNN-derived embeddings from multi-omics may have different meanings. To obtain similar embeddings from multi-omics, we aligned the embeddings among modalities by maximizing their correlations before integration. This process also ensured that each dimension of the embeddings from multi-omics had the same meaning. For samples with complete multi-omics data, we calculated the Pearson correlation between the two modality embeddings for each embedding dimension, and the similarity was calculated as the average of absolute Pearson correlations across embedding dimensions. For samples with incomplete multi-omics data (i.e., having only transcriptomic or proteomic measurements), we calculated the Pearson correlation for each pair of samples with the same class labels but different modalities, and the similarity was calculated as the average of absolute Pearson correlations across all possible sample pairs. The sum of the negative averages was included in the regularization loss terms.

#### Integration using set transformers

Let ${{\boldsymbol{z}}}_{i}\in {{\mathbb{R}}}^{m}$ be the GNN embeddings corresponding to the ${i}^{{th}}$ ‘omics modality. We collected embeddings from GNNs corresponding to each individual modality into a set ${\boldsymbol{Z}}={\left({{\boldsymbol{z}}}_{0},{{\boldsymbol{z}}}_{1},\cdots ,{{\boldsymbol{z}}}_{K}\right)}^{T}\in {{\mathbb{R}}}^{(K+1)\times m}$, where $K\left(\ge 1\right)$ was the number of modalities and ${{\boldsymbol{z}}}_{o}\in {{\mathbb{R}}}^{m}$ was a set of learnable parameters called the *class token*. We then used the standard transformer encoder architecture described in ref. ^64^ to set up an integrative classifier. The prediction from the integrative classifier, $\hat{y}\in {{\mathbb{R}}}^{C}$ (where $C$ was the number of classes) was given by -

2 $$\hat{y}={MLP}({Encoder}{({\boldsymbol{Z}})}_{0}),$$

fweights during training was randomizedflinearly weight node featureswhere ${Encoder}{\left({\boldsymbol{Z}}\right)}_{0}\in {{\mathbb{R}}}^{m}$ was the latent representation of the learnable input class token ${{\boldsymbol{z}}}_{o}$, and ${MLP}\left(\cdot \right):{{\mathbb{R}}}^{m}\to {{\mathbb{R}}}^{C}$ was a fully connected neural network which mapped the final class token representation to the target label space. The transformer encoder was a composition of $n$ encoder blocks i.e., $Encoder({\boldsymbol{Z}})={E}_{n}\,\,{\atop{\circ }}E_{n-1}\ldots \,{\atop{\circ }}\,E_{1}({\boldsymbol{Z}})$. The sequence of operations within each encoder block was as follows:

1. Multi-head self-attention (MSA), residual connection, and layer normalization (LN)^65^ on the set of ‘omics tokens - ${{\boldsymbol{Z}}}^{{\boldsymbol{{\prime} }}}=LN({\boldsymbol{Z}}+MSA({\boldsymbol{Z}})),$
2. Position-specific feedforward network followed by a residual connection and layer normalization (LN) - ${{\boldsymbol{Z}}}_{{out}}={LN}\left({{\boldsymbol{Z}}}^{{\prime} }+{FFN}\left({{\boldsymbol{Z}}}^{{\prime} }\right)\right),$ where ${FFN}\left(\cdot \right):{{\mathbb{R}}}^{m}\to {{\mathbb{R}}}^{m}$ was a one hidden layer MLP with ReLU activation and operated on each token individually, i.e., $FFN({\boldsymbol{Z}})=FFN({{\boldsymbol{z}}}_{0},{{\boldsymbol{z}}}_{1},\cdots ,{{\boldsymbol{z}}}_{K})=(FFN{({{\boldsymbol{z}}}_{0}),FFN({{\boldsymbol{z}}}_{1}),\ldots ,FFN({{\boldsymbol{z}}}_{K}))}^{T}).$

#### Full model architecture and training

Our full integrative multi-omics model was expressed as:

3 $$y=h{(g}_{1}\left({{\boldsymbol{x}}}_{1},{{\mathcal{E}}}_{1},{{\mathcal{W}}}_{1}\right),{g}_{2}\left({{\boldsymbol{x}}}_{2},{{\mathcal{E}}}_{2},{{\mathcal{W}}}_{2}\right),\ldots ,{g}_{K}\left({{\boldsymbol{x}}}_{K},{{\mathcal{E}}}_{K},{{\mathcal{W}}}_{K}\right)),$$

where, $h\left(\cdot \right)$ was a set transformer which integrated feature representations generated by the ‘omics GNNs ${g}_{i}s$, ${{\boldsymbol{x}}}_{i}s$ were the node features for the ${i}^{{th}}$ ‘omics modality and ${{\mathcal{E}}}_{i},{{\mathcal{W}}}_{i}$ were the edge index list and edge attribute list associated with the ${i}^{{th}}$ modality. A schematic of the architecture is shown in Fig. 1. Given multi-omics data for a given sample, measurements for each modality were processed through their respective GNN modules. The parameters of the GNN were trained by classifying the embeddings to the corresponding target labels using multi-layer perceptrons (MLP) or a set transformer, which collected embeddings for all available modalities, integrated them, and then made a prediction on the target label.

#### Training with complete samples

We first describe the training of our model when the multi-omics data set had complete samples, i.e., we had measurements in all available ‘omics modalities for all patients. We adopted three-fold cross-validation to evaluate the predictive accuracy of our model, namely we randomly split the total dataset into three folds, maintaining the ratio of sample labels in each fold as in the whole dataset. Samples from two of these splits were chosen as training samples, while samples from the remaining split were used as validation samples. This procedure was repeated three times, hence each split had a chance to serve as validation samples. Since samples in our dataset had binarized labels (AD/control), we therefore used the binary cross entropy loss function. We set up our end-to-end integrative model as detailed in the previous sections and trained it using stochastic gradient descent optimization, specifically the Adam optimization method^66^, using mini-batches of training data. The objective function we optimized was as follows:

4 $${\mathcal{L}}=\mathop{\sum }\limits_{i=1}^{K}{{\mathcal{L}}}_{i}+{{\lambda }_{1}{\mathcal{L}}}_{\mathrm{int}}+{\lambda }_{2}\sum _{{\rm{i}} > {\rm{j}}}{{\mathcal{L}}}_{i,j}^{\text{align}}+{{\lambda }_{3}{\mathcal{L}}}_{\text{reg}},$$

where ${{\mathcal{L}}}_{i}$ was the loss incurred on the predictions made by the MLP classifier on the embeddings of the ${i}^{{th}}$ modality, ${{\mathcal{L}}}_{\mathrm{int}}$ was the loss incurred on the predictions made by the integrative module, i.e., the set transformer, ${{\mathcal{L}}}_{i,j}^{\text{align}}$ was the alignment loss between the ${i}^{{th}}$ and ${j}^{{th}}$ modalities, and ${{\mathcal{L}}}_{\text{reg}}$ were norm penalties on the learnable parameters in the model. ${{\lambda }_{1},\lambda }_{2},,\,{\lambda }_{3}$ were trade-off hyper-parameters. Furthermore, we applied sample weights on our prediction losses (${{\mathcal{L}}}_{i}$ and ${{\mathcal{L}}}_{\mathrm{int}}$) to account for any potential class imbalance.

#### Training with incomplete samples

To account for incomplete samples (i.e., samples with missing measurements in one or more modalities), we split our total training dataset into disjoint subsets based on modality representation. For our transcriptomics and proteomics datasets, we split the total dataset into three subsets—the set of common samples and two additional sets comprising samples having measurements in transcriptomics or proteomics alone. Each epoch of training for our integrative model now comprises a single epoch through each disjoint subset. The common sample subset was trained with the full loss function as described in the previous section. The loss function was modified for training data subsets with missing modalities. For a subset of the training data with measurements in a single modality only, the components ${{\mathscr{L}}}_{\mathrm{int}}$ and ${{\mathcal{L}}}_{i,j}^{\text{align}}$ were set to 0. The order in which the disjoint data subsets were processed to update the model weights during training was randomized from epoch to epoch.

#### Training time

Training time was dependent on both the number of nodes in a graph and the training sample size. Runtime was benchmarked using CPU-only and GPU-accelerated model training, with each 200-epoch model training iteration being run three times, each using two-thirds of the total samples as training samples. Quoted are the mean and standard deviation of those three training iterations. For CPU-only results, the models were trained using a single 2.7 GHz CPU core. The runtime ranged from 10 m 2s ± 6 s for the biodomain with the smallest number of nodes (Tau Homeostasis) to 10 h 1 m 58 s ± 5 m 42 s for the biodomain with the largest number of nodes (Proteostatis). For GPU-accelerated results, the models were trained using a single 2.7 GHz CPU core aided by an NVIDIA Tesla V100 GPU. The runtime ranged from 5 m 42 s ± 34 s (Tau Homeostasis) to 16 m 50 s ± 3 s (Proteostasis).

#### Hyperparameter selection

As described in the previous section, we created -three stratified splits of our dataset, from which we picked one fold for validation and used the remaining two for training. For any given combination of hyperparameters, we trained our models 3 times per split, each with a different random model weight initialization, and cross-validated on the validation dataset. In total, we trained the model 9 times with the same hyperparameters. We averaged the validation performance of our model over these 9 trials and reported it as the predictive performance corresponding to a given setting of hyperparameters. We performed a grid search over hyperparameters and picked the hyperparameters with the highest average validation accuracy over 9 trials.

For GNNRAI training, we conducted a non-exhaustive preliminary hyperparameter search, using 3 of the 16 biodomain datasets (autophagy, myelination, and cell cycle), to establish baseline ranges for all hyperparameters and identify those to which model performance was most sensitive. During final model training we performed a smaller hyperparameter grid search over the set of critical hyperparameters identified during the preliminary stage. A summary of the hyperparameters explored during the final model training stage, along with a selection of the most critical fixed hyperparameters, are shown in Supplementary Table 1.

#### Integrated gradients

Let ${\boldsymbol{x}}={\left({x}_{1},{x}_{2},\ldots ,{x}_{d}\right)}^{{\rm{T}}}\in {{\mathbb{R}}}^{d}$ be the input features to a deep learning model, $f:{{\mathbb{R}}}^{d}\to {{\mathbb{R}}}^{C}$, where $C$ was the number of classes of the target label. Let ${f}_{c}\left({\boldsymbol{x}}\right)$ be the model output score for the ${c}^{{th}}$ class. The components of the gradient vector, ${\nabla }_{x}{f}_{c}$, represented the sensitivity of the class score to small perturbations of the input features, and the magnitude of its components may be interpreted as a proxy for the importance of input features. The integrated gradient attribution of the ${i}^{{th}}$ input feature on the ${c}^{{th}}$ class was defined as follows:

5 $${\phi }_{i}=\left({x}_{i}-{x}_{{i}^{{\prime} }}\right)\times {\int }_{\alpha =0}^{1}\frac{{\rm{\partial }}{f}_{c}\left({x}^{{\prime} }+\alpha (x-{x}^{{\prime} })\right)}{{\rm{\partial }}{x}_{i}}\text{d}\alpha ,$$

where $x{\prime}$ was a user-defined baseline input. The integrand represented the model evaluation at an input constructed by the linear interpolation between the baseline ${x}^{{\prime} }$ and the true input $x$. The choice of the baseline was task-dependent. For instance, in image classification tasks, where the input $x$ is a tensor representing pixel intensities, it is customary to pick the zero tensor as the baseline, i.e., ${x}^{{\prime} }=0$. In our multi-omics model, inputs were node features representing gene expression/protein abundance levels. We set our baseline as the average expression/abundance level in our training control samples. Features with large magnitude attribution scores on the disease class label (i.e., class label 1) were implicated as informative disease biomarkers.

#### Integrated Hessians

Integrated Hessians for pairwise feature interactions^18^ is a natural extension of the method of integrated gradients. Let ${{\rm{\phi }}}_{i}:{{\mathbb{R}}}^{d}\to {\mathbb{R}}$ be the integrated gradient attributions on the ${i}^{{th}}$ input feature. Applying integrated gradient explanations on the function ${{\rm{\phi }}}_{i}\left({\boldsymbol{x}}\right)$ resulted in a new $d$-dimensional vector whose ${j}^{{th}}$ component ${\varGamma }_{i,j}\left({\boldsymbol{x}}\right)={{\rm{\phi }}}_{j}\left({{\rm{\phi }}}_{i}\left({\boldsymbol{x}}\right)\right)$ explained the contribution of feature ${x}_{j}$ to the model attributions on feature ${x}_{i}$. We interpreted the quantity ${\varGamma }_{i,j}\left({\boldsymbol{x}}\right)$ as the interaction between features $i$ and $j$. For $i\,\ne \,j$, the feature interaction scores were given by:

6 $${\varGamma }_{i,j}(x)=\left({x}_{i}-{{x}_{i}}^{{\rm{{\prime} }}}\right)\left({x}_{j}-{x}_{j}{\rm{{\prime} }}\right)\times {\int }_{\alpha =0}^{1}{\int }_{\beta =0}^{1}\alpha \beta \frac{{{\rm{\partial }}}^{2}{f}_{c}\left({x}^{{\rm{{\prime} }}}+\alpha \beta ({\rm{x}}-{{\rm{x}}}^{{\rm{{\prime} }}})\right)}{{\rm{\partial }}{x}_{i}{\rm{\partial }}{x}_{j}}\text{d}\alpha \text{d}\beta ,$$

where ${x}^{{\prime} }$ was a baseline input. The self-interaction term ${\varGamma }_{i,i}\left(x\right)$ was given by:

7 $${\varGamma }_{i,i}\left(x\right)={{\rm{\phi }}}_{i}\left(x\right)-\sum _{i\ne j}{\varGamma }_{i,j}\left(x\right),$$

where the ${i}^{{th}}$ feature integrated score ${{\rm{\phi }}}_{i}\left(x\right)$ represented the marginal contribution of ${x}_{i}$ to model prediction. The self-interaction score ${\Gamma }_{i,i}\left(x\right)$ was, thus, defined as the difference between the marginal contribution of ${x}_{i}$ and every pairwise interaction involving ${x}_{i}$.

### Identify informative markers or marker interactions with specified false discovery rate (FDR)

To determine the importance score threshold above which a marker or a marker interaction was viewed as informative, we adopted the permutation approach to compute the empirical FDR. Specifically, we randomly permuted the order of the ground truth labels to generate $B$ permuted datasets of ground truth labels. We then trained the GNN model on each of the $B$ permuted datasets and computed importance scores for markers using integrated gradients or marker interactions using integrated Hessians. Following the non-parametric procedure outlined in ref. ^67^, we calculated the false discovery rate for a given threshold $d$ as:

8 $${FDR}\left(d\right)={\pi }_{0}\frac{\left({\sum }_{b=1}^{B}\frac{\left|\left\{i:{{\rm{\phi }}}_{i}^{(b)} > d\right\}\right|}{B}\right)}{\left|\left\{i:{{\rm{\phi }}}_{i} > d\right\}\right|},$$

where, ${{\rm{\phi }}}_{i}$ was the importance score of marker or interaction $i$ in the unpermuted data, ${{\rm{\phi }}}_{i}^{(b)}$ was the importance score of marker or interaction $i$ in the ${b}^{{th}}$ permutation, ${\pi }_{0}$ was the prior probability that a marker or an interaction was uninformative. For AD study, ${\pi }_{0}$ is close to 1, and we took the value of 0.97. The scores obtained under the null hypothesis (permuted data) could be used by different analyses from the same dataset – for instance, analyses from different random initializations and different training dataset folds. For an FDR threshold of $\le$0.05, the estimated empirical FDR was confidently accurate for ≥100 permutations^24^. We performed 300 permutations in all our experiments.

#### MOGONET model structure and training

We followed the same architecture for MOGONET as described in ref. ^12^—three graph convolutional network (GCN) layers followed by a view correlation discovery network (VCDN). We also followed the same training procedure as specified in ref. ^12^—‘Omics specific GCN feature extractors are pretrained initially, and a secondary end-to-end training stage is performed with the GCN and VCDN networks combined’. A summary of the hyperparameter settings swept through for MOGONET training is shown in Supplementary Table 2^68^.

## Supplementary information

Supplementary Information

## Data Availability

All the data analyzed in the article were downloaded from the AD Knowledge Portal https://adknowledgeportal.synapse.org^68^. The ROSMAP, MSBB, and Mayo bulk brain RANSeq data were from https://www.synapse.org/Synapse:syn30821562; the ROSMAP bulk brain TMT proteomics data were from https://www.synapse.org/Synapse:syn31534849; and the MSBB bulk brain TMT proteomics data were from https://www.synapse.org/Synapse:syn52331749. Alzheimer’s disease biodomain network files can be found at https://www.synapse.org/Synapse:syn51739831.

## Code availability

The code is available at https://github.com/Yi-Li/code-GNN-multi-omics.

## References

[1] Schneider, M. V. & Orchard, S. Omics technologies, data and bioinformatics principles. *Methods Mol. Biol.* **719**, 3–30 (2011). 21370077 10.1007/978-1-61779-027-0_1

[2] Hrdlickova, R., Toloue, M. & Tian, B. RNA-Seq methods for transcriptome analysis, *Wiley Interdiscip. Rev. RNA*10.1002/wrna.1364 (2017). 10.1002/wrna.1364 PMC5717752 27198714

[3] Chen, Y. R., Yu, S. & Zhong, S. Profiling DNA methylation using bisulfite sequencing (BS-Seq). *Methods Mol. Biol.* **1675**, 31–43 (2018). 29052183 10.1007/978-1-4939-7318-7_2

[4] Gunther, O. P. et al. A computational pipeline for the development of multi-marker bio-signature panels and ensemble classifiers. *BMC Bioinforma.* **13**, 326 (2012). 10.1186/1471-2105-13-326 PMC3575305 23216969

[5] Argelaguet, R. et al. Multi-Omics Factor Analysis—a framework for unsupervised integration of multi-omics data sets. *Mol. Syst. Biol.* **14**, e8124 (2018). 29925568 10.15252/msb.20178124 PMC6010767

[6] Shen, R., Olshen, A. B. & Ladanyi, M. Integrative clustering of multiple genomic data types using a joint latent variable model with application to breast and lung cancer subtype analysis. *Bioinformatics* **25**, 2906–2912 (2009). 19759197 10.1093/bioinformatics/btp543 PMC2800366

[7] Gao, C. et al. Iterative single-cell multi-omic integration using online learning. *Nat. Biotechnol.* **39**, 1000–1007 (2021). 33875866 10.1038/s41587-021-00867-x PMC8355612

[8] Wang, B. et al. Similarity network fusion for aggregating data types on a genomic scale. *Nat. Methods* **11**, 333–337 (2014). 24464287 10.1038/nmeth.2810

[9] Speicher, N. K. & Pfeifer, N. Integrating different data types by regularized unsupervised multiple kernel learning with application to cancer subtype discovery. *Bioinformatics* **31**, i268–i275 (2015). 26072491 10.1093/bioinformatics/btv244 PMC4765854

[10] Mariette, J. & Villa-Vialaneix, N. Unsupervised multiple kernel learning for heterogeneous data integration. *Bioinformatics* **34**, 1009–1015 (2018). 29077792 10.1093/bioinformatics/btx682

[11] Vahabi, N. & Michailidis, G. Unsupervised Multi-Omics data integration methods: a comprehensive review. *Front. Genet.* **13**, 854752 (2022). 35391796 10.3389/fgene.2022.854752 PMC8981526

[12] Wang, T. et al. MOGONET integrates multi-omics data using graph convolutional networks allowing patient classification and biomarker identification. *Nat. Commun.* **12**, 3445 (2021). 34103512 10.1038/s41467-021-23774-w PMC8187432

[13] Li, X. et al. MoGCN: A multi-omics integration method based on graph convolutional network for cancer subtype analysis. *Front. Genet.* **13**, 806842 (2022). 35186034 10.3389/fgene.2022.806842 PMC8847688

[14] Cary, G. A. et al. Genetic and multi-omic risk assessment of Alzheimer’s disease implicates core associated biological domains. *Alzheimers Dement. (N. Y)* **10**, e12461 (2024). 38650747 10.1002/trc2.12461 PMC11033838

[15] Lee, J. et al. Set transformer: a framework for attention-based permutation-invariant neural networks. in *International Conference on Machine Learning*, 2019: PMLR, 3744–3753.

[16] Yang, M. et al. Multi-omic integration via similarity network fusion to detect molecular subtypes of ageing. *Brain Commun.* **5**, fcad110 (2023). 37082508 10.1093/braincomms/fcad110 PMC10110975

[17] Sundararajan, M., Taly, A. & Yan, Q. Axiomatic attribution for deep networks. in *International Conference on Machine Learning*, 2017: PMLR, 3319–3328.

[18] Janizek, J. D., Sturmfels, P. & Lee, S.-I. Explaining explanations: axiomatic feature interactions for deep networks. *J. Mach. Learn. Res.* **22**, 1–54 (2021).

[19] Cerami, E. G. et al. Pathway Commons, a web resource for biological pathway data. *Nucleic Acids Res.* **39**, D685–D690 (2011). 21071392 10.1093/nar/gkq1039 PMC3013659

[20] Johnson, E. C. B. et al. Large-scale deep multi-layer analysis of Alzheimer’s disease brain reveals strong proteomic disease-related changes not observed at the RNA level. *Nat. Neurosci.* **25**, 213–225 (2022). 35115731 10.1038/s41593-021-00999-y PMC8825285

[21] Wang, M. et al. The Mount Sinai cohort of large-scale genomic, transcriptomic and proteomic data in Alzheimer’s disease. *Sci. Data* **5**, 180185 (2018). 30204156 10.1038/sdata.2018.185 PMC6132187

[22] Allen, M. et al. Human whole genome genotype and transcriptome data for Alzheimer’s and other neurodegenerative diseases. *Sci. Data* **3**, 160089 (2016). 27727239 10.1038/sdata.2016.89 PMC5058336

[23] McKhann, G. et al. Clinical diagnosis of Alzheimer’s disease: report of the NINCDS-ADRDA Work Group under the auspices of Department of Health and Human Services Task Force on Alzheimer’s Disease. *Neurology* **34**, 939–944 (1984). 6610841 10.1212/wnl.34.7.939

[24] Millstein, J. & Volfson, D. Computationally efficient permutation-based confidence interval estimation for tail-area FDR. *Front. Genet.* **4**, 179 (2013). 24062767 10.3389/fgene.2013.00179 PMC3775454

[25] Drummond, E. et al. The amyloid plaque proteome in early onset Alzheimer’s disease and Down syndrome. *Acta Neuropathol. Commun.* **10**, 53 (2022). 35418158 10.1186/s40478-022-01356-1 PMC9008934

[26] Levites, Y. et al. Abeta amyloid scaffolds the accumulation of matrisome and additional proteins in Alzheimer’s disease. *bioRxiv*10.1101/2023.11.29.568318 (2023).

[27] Tandon, R., Levey, A. I., Lah, J. J., Seyfried, N. T. & Mitchell, C. S. Machine learning selection of most predictive brain proteins suggests role of sugar metabolism in Alzheimer’s disease. *J. Alzheimers Dis.* **92**, 411–424 (2023). 36776048 10.3233/JAD-220683 PMC10041447

[28] Watson, C. M. et al. Quantitative mass spectrometry analysis of cerebrospinal fluid protein biomarkers in Alzheimer’s disease. *Sci. Data* **10**, 261 (2023). 37160957 10.1038/s41597-023-02158-3 PMC10170100

[29] Beckmann, N. D. et al. Multiscale causal networks identify VGF as a key regulator of Alzheimer’s disease. *Nat. Commun.* **11**, 3942 (2020). 32770063 10.1038/s41467-020-17405-z PMC7414858

[30] Serrano-Pozo, A., Das, S. & Hyman, B. T. APOE and Alzheimer’s disease: advances in genetics, pathophysiology, and therapeutic approaches. *Lancet Neurol.* **20**, 68–80 (2021). 33340485 10.1016/S1474-4422(20)30412-9 PMC8096522

[31] Arboleda-Velasquez, J. F. et al. Resistance to autosomal dominant Alzheimer’s disease in an APOE3 Christchurch homozygote: a case report. *Nat. Med.* **25**, 1680–1683 (2019). 31686034 10.1038/s41591-019-0611-3 PMC6898984

[32] Martens, Y. A. et al. ApoE Cascade Hypothesis in the pathogenesis of Alzheimer’s disease and related dementias. *Neuron* **110**, 1304–1317 (2022). 35298921 10.1016/j.neuron.2022.03.004 PMC9035117

[33] Tsatsanis, A. et al. The acute phase protein lactoferrin is a key feature of Alzheimer’s disease and predictor of Abeta burden through induction of APP amyloidogenic processing. *Mol. Psychiatry* **26**, 5516–5531 (2021). 34400772 10.1038/s41380-021-01248-1 PMC8758478

[34] Zhang, Z. et al. Cleavage of tau by asparagine endopeptidase mediates the neurofibrillary pathology in Alzheimer’s disease. *Nat. Med.* **20**, 1254–1262 (2014). 25326800 10.1038/nm.3700 PMC4224595

[35] Yao, Y. et al. A delta-secretase-truncated APP fragment activates CEBPB, mediating Alzheimer’s disease pathologies. *Brain* **144**, 1833–1852 (2021). 33880508 10.1093/brain/awab062 PMC8320270

[36] Wang, S. et al. IQGAP3, a novel effector of Rac1 and Cdc42, regulates neurite outgrowth. *J. Cell Sci.* **120**, 567–577 (2007). 17244649 10.1242/jcs.03356

[37] Bellenguez, C. et al. New insights into the genetic etiology of Alzheimer’s disease and related dementias. *Nat. Genet.* **54**, 412–436 (2022). 35379992 10.1038/s41588-022-01024-z PMC9005347

[38] Ehehalt, R., Keller, P., Haass, C., Thiele, C. & Simons, K. Amyloidogenic processing of the Alzheimer beta-amyloid precursor protein depends on lipid rafts. *J. Cell Biol.* **160**, 113–123 (2003). 12515826 10.1083/jcb.200207113 PMC2172747

[39] Baloni, P. et al. Multi-Omic analyses characterize the ceramide/sphingomyelin pathway as a therapeutic target in Alzheimer’s disease. *Commun. Biol.* **5**, 1074 (2022). 36209301 10.1038/s42003-022-04011-6 PMC9547905

[40] R. et al. Comparative brain metabolomics reveals shared and distinct metabolic alterations in Alzheimer’s disease and progressive supranuclear palsy. *medRxiv*10.1101/2023.07.25.23293055 (2023). 10.1002/alz.14249 PMC11667510 39439201

[41] Yan, H. F. et al. Ferroptosis: mechanisms and links with diseases. *Signal Transduct. Target Ther.* **6**, 49 (2021). 33536413 10.1038/s41392-020-00428-9 PMC7858612

[42] Wang, F. et al. Iron dyshomeostasis and ferroptosis: a new Alzheimer’s disease hypothesis?. *Front Aging Neurosci.* **14**, 830569 (2022). 35391749 10.3389/fnagi.2022.830569 PMC8981915

[43] Chen, C. et al. Legumain promotes tubular ferroptosis by facilitating chaperone-mediated autophagy of GPX4 in AKI. *Cell Death Dis.* **12**, 65 (2021). 33431801 10.1038/s41419-020-03362-4 PMC7801434

[44] Yan, L. et al. Integrative analysis of TBI data reveals Lgmn as a key player in immune cell-mediated ferroptosis. *BMC Genomics* **24**, 747 (2023). 38057699 10.1186/s12864-023-09842-z PMC10699068

[45] Wang, Y., Liu, Y., Liu, J., Kang, R. & Tang, D. NEDD4L-mediated LTF protein degradation limits ferroptosis. *Biochem. Biophys. Res. Commun.* **531**, 581–587 (2020). 32811647 10.1016/j.bbrc.2020.07.032

[46] Xiao, Z. et al. Reduction of lactoferrin aggravates neuronal ferroptosis after intracerebral hemorrhagic stroke in hyperglycemic mice. *Redox Biol.* **50**, 102256 (2022). 35131600 10.1016/j.redox.2022.102256 PMC8829351

[47] Liu, T., Jiang, L., Tavana, O. & Gu, W. The deubiquitylase OTUB1 mediates ferroptosis via stabilization of SLC7A11. *Cancer Res.* **79**(Apr 15), 1913–1924 (2019). 30709928 10.1158/0008-5472.CAN-18-3037 PMC6467774

[48] Ye, H. et al. Involvement of CD44 and MAPK14-mediated ferroptosis in hemorrhagic shock. *Apoptosis* **29**, 154–168 (2024). 37751106 10.1007/s10495-023-01894-6

[49] Lv, T. et al. Sevoflurane causes neurotoxicity and cognitive impairment by regulating Hippo signaling pathway-mediated ferroptosis via upregulating PRKCD. *Exp. Neurol.* **377**, 114804 (2024). 38704083 10.1016/j.expneurol.2024.114804

[50] Xia, L. & Gong, N. Identification and verification of ferroptosis-related genes in the synovial tissue of osteoarthritis using bioinformatics analysis. *Front. Mol. Biosci.* **9**, 992044 (2022). 36106017 10.3389/fmolb.2022.992044 PMC9465169

[51] Fang, W., Liu, J., Zhang, F., Pang, C. & Li, X. A novel cholesterol metabolism-related ferroptosis pathway in hepatocellular carcinoma. *Discov. Oncol.* **15**, 7 (2024). 38191842 10.1007/s12672-023-00822-z PMC10774324

[52] Lian, K. et al. Hub genes, a diagnostic model, and immune infiltration based on ferroptosis-linked genes in schizophrenia. *IBRO Neurosci. Rep.* **16**, 317–328 (2024). 38390236 10.1016/j.ibneur.2024.01.007 PMC10882140

[53] Xie, J. et al. Identification and analysis of biomarkers associated with oxidative stress and ferroptosis in recurrent miscarriage. *Medicines* **103**, e38875 (2024). 10.1097/MD.0000000000038875 PMC11398789 39029052

[54] Zhao, S. T. et al. Exploring the molecular biology of ischemic cardiomyopathy based on ferroptosis‑related genes. *Exp. Ther. Med.* **27**, 221 (2024). 38590563 10.3892/etm.2024.12509 PMC11000445

[55] Ying, Z., Bourgeois, D., You, J., Zitnik, M. & Leskovec, J. Gnnexplainer: generating explanations for graph neural networks. *Adv. Neural Inf. Process. Syst.* **32**, 9244–9255 (2019). PMC7138248 32265580

[56] Folstein, M. F., Folstein, S. E. & McHugh, P. R. “Mini-mental state”: a practical method for grading the cognitive state of patients for the clinician. *J. Psychiatr. Res.* **12**, 189–198 (1975). 1202204 10.1016/0022-3956(75)90026-6

[57] Braak, H., Alafuzoff, I., Arzberger, T., Kretzschmar, H. & Del Tredici, K. Staging of Alzheimer disease-associated neurofibrillary pathology using paraffin sections and immunocytochemistry. *Acta Neuropathol.* **112**, 389–404 (2006). 16906426 10.1007/s00401-006-0127-z PMC3906709

[58] Wolfsgruber, S. et al. The CERAD neuropsychological assessment battery total score detects and predicts Alzheimer disease dementia with high diagnostic accuracy. *Am. J. Geriatr. Psychiatry* **22**, 1017–1028 (2014). 23759289 10.1016/j.jagp.2012.08.021

[59] Ashburner, M. et al. Gene ontology: tool for the unification of biology. The Gene Ontology Consortium. *Nat. Genet.* **25**, 25–29 (2000). 10802651 10.1038/75556 PMC3037419

[60] Cangea, C., Veličković, P., Jovanović, N., Kipf, T. & Liò, P. Towards sparse hierarchical graph classifiers. PReprint at *arXiv*10.48550/arXiv.1811.01287 (2018).

[61] Ahmadi, A. H. K. *Memory-Based Graph Networks*. (University of Toronto, Canada, 2020).

[62] Li, X. et al. BrainGNN: interpretable Brain Graph Neural Network for fMRI analysis. *Med. Image Anal.* **74**, 102233 (2021). 34655865 10.1016/j.media.2021.102233 PMC9916535

[63] Kipf, T. N. & Welling, M. Semi-supervised classification with graph convolutional networks. Preprint at *arXiv*10.48550/arXiv.1609.02907 (2016).

[64] Vaswani, A. Attention is all you need. *Adv. Neural Inf. Process. Syst.* 6000–6010 10.48550/arXiv.1706.03762 (2017).

[65] Ba, J. Lei, Kiros, R. & Hinton, G. E. Layer normalization. Preprint at *arXiv*10.48550/arXiv.1607.06450 (2016).

[66] Kingma, D. P. Adam: a method for stochastic optimization. Preprint at *arXiv*10.48550/arXiv.1412.6980 (2014).

[67] Xie, Y., Pan, W. & Khodursky, A. B. A note on using permutation-based false discovery rate estimates to compare different analysis methods for microarray data. *Bioinformatics* **21**, 4280–4288 (2005). 16188930 10.1093/bioinformatics/bti685

[68] Greenwood, A. K. et al. The AD knowledge portal: a repository for Multi-Omic Data on Alzheimer’s disease and aging. *Curr. Protoc. Hum. Genet* **108**, e105 (2020). 33085189 10.1002/cphg.105 PMC7587039

