# Supplementary material for: Effective integration of multi-omics with prior knowledge to identify biomarkers via explainable graph neural networks
Source: NPJ Syst Biol Appl. 2025 May 8;11:43. doi: 10.1038/s41540-025-00519-9 (PMC12062277; doi:10.1038/s41540-025-00519-9)
Supplement: Supplementary file 1 — Supplementary Information [file 41540_2025_519_MOESM1_ESM.pdf]

Effective integration of multi-omics with prior knowledge to identify biomarkers via explainable graph neural networks

Supplementary Table 1: Summary of scanned and critical fixed hyperparameters in GNNRAI training.

**Hyperparameter**	**Values**
Graph embedding dimension	16
Batch size	16
Learning rate	2.5×10^{-3}
Number of clusters in memory pooling layer	40, 80
L1 regularization penalty in classifier	0, 1×10^{-3}
L2 regularization penalty in classifier	0, 1×10^{-3}

Supplementary Table 2. Hyperparameters swept through for tuning the benchmark MOGONET model.

**Hyperparameter**	**Values**
Number of edges per node, k	2, 3
Number of hidden units in 1st two GCN layers, h_1	200, 400
Number of hidden units in final GCN layer, h_2	100, 200
Pretraining learning rate	10^{-4}, 10^{-5}
Learning rate for GCN layers during end-to-end training stage	5×10^{-4}, 5×10^{-5}
Learning rate for VCDN layer during end-to-end training stage	1×10^{-3}, 1×10^{-4}

Supplementary Table 3. Validation performance of our proposed integrative model compared to validation performance of the benchmark MOGONET model on the set of common samples having both proteomics and transcriptomics measurements. The performance of the integrative models is also compared to unimodal GNN transcriptomics and proteomics models. This data was used to generate Figure 2.

Biodomain Name	Biodomain Abbreviation	Unimodal (RNA)	Unimodal (Protein)	Proposed Multimodal	MOGONET
Apoptosis	apo	0.724	0.832	0.848	0.863
APP Metabolism	app	0.694	0.845	0.865	0.852
Autophagy	aut	0.725	0.75	0.803	0.753
Cell Cycle	cel	0.75	0.817	0.82	0.808
Endolysosome	end	0.722	0.819	0.845	0.8
Immune Response	imm	0.741	0.813	0.833	0.788
Lipid Metabolism	lip	0.741	0.813	0.846	0.806
Metal Binding	met	0.753	0.789	0.823	0.753
Mitochondrial Metabolism	mit	0.738	0.803	0.833	0.819
Myelination	mye	0.763	0.855	0.877	0.864
Oxidative Stress	oxi	0.743	0.797	0.827	0.787
Proteostasis	pro	0.727	0.82	0.822	0.794
Structural Stabilization	str	0.744	0.854	0.861	0.86
Synapse	syn	0.763	0.846	0.873	0.867
Tau Homeostasis	tau	0.709	0.82	0.846	0.848
Vasculature	vas	0.741	0.814	0.846	0.851

Supplementary Table 4. Validation performance of the integrative multimodal model compared to performance of unimodal GNN models trained on the incomplete multi-omics datasets for 16 AD biodomains. The performance of the multimodal model is calculated on two sets of validation samples – the set of all validation samples with transcriptomics measurements and that with proteomics measurements. This data was used to generate Figure 3.

Biodomain Name	Biodomain Abbreviation	Unimodal (RNA)	Unimodal (Protein)	Integrated (RNA)	Integrated (Protein)
Apoptosis	apo	0.787	0.822	0.829	0.847
APP Metabolism	app	0.764	0.839	0.816	0.851
Autophagy	aut	0.769	0.791	0.787	0.812
Cell Cycle	cel	0.783	0.786	0.809	0.822
Endolysosome	end	0.793	0.828	0.825	0.844
Immune Response	imm	0.789	0.801	0.822	0.831
Lipid Metabolism	lip	0.802	0.81	0.827	0.822
Metal Binding	met	0.791	0.795	0.806	0.815
Mitochondrial Metabolism	mit	0.785	0.791	0.81	0.826
Myelination	mye	0.781	0.846	0.837	0.874
Oxidative Stress	oxi	0.777	0.788	0.803	0.818
Proteostasis	pro	0.79	0.805	0.818	0.82
Structural Stabilization	str	0.794	0.851	0.827	0.853
Synapse	syn	0.788	0.857	0.823	0.863
Tau Homeostasis	tau	0.717	0.832	0.768	0.848
Vasculature	vas	0.802	0.822	0.832	0.852

Supplementary Table 5. Predictive performance of applying unimodal transcriptomics model trained on ROSMAP DLPFC training dataset to validation transcriptomics samples from various cohorts and brain tissues. This data was used to generate Figure 4.

Biodomain Name	Biodomain Abbreviation	ROSMAP (DLPFC)	ROSMAP (ACC)	ROSMAP (PCC)	MSBB (PHG)	MSBB (FP)	MSBB (IFG)	MSBB (STG)	Mayo (TCX)
Apoptosis	apo	0.787	0.684	0.824	0.865	0.8	0.844	0.824	0.767
APP Metabolism	app	0.764	0.678	0.809	0.847	0.751	0.841	0.769	0.742
Autophagy	aut	0.769	0.69	0.815	0.841	0.782	0.805	0.803	0.71
Cell Cycle	cel	0.783	0.68	0.812	0.837	0.773	0.827	0.824	0.719
Endolysosome	end	0.793	0.684	0.805	0.804	0.721	0.804	0.8	0.731
Immune Response	imm	0.789	0.683	0.837	0.862	0.828	0.864	0.865	0.792
Lipid Metabolism	lip	0.802	0.705	0.844	0.863	0.82	0.862	0.826	0.772
Metal Binding	met	0.791	0.671	0.835	0.836	0.818	0.801	0.809	0.694
Mitochondrial Metabolism	mit	0.785	0.716	0.833	0.838	0.77	0.822	0.79	0.776
Myelination	mye	0.781	0.661	0.777	0.81	0.804	0.805	0.8	0.722
Oxidative Stress	oxi	0.777	0.679	0.802	0.781	0.784	0.816	0.779	0.754
Proteostasis	pro	0.79	0.71	0.831	0.864	0.803	0.851	0.833	0.735
Structural Stabilization	str	0.794	0.691	0.831	0.882	0.817	0.879	0.846	0.824
Synapse	syn	0.788	0.689	0.83	0.882	0.807	0.869	0.803	0.772
Tau Homeostasis	tau	0.717	0.663	0.708	0.712	0.698	0.717	0.684	0.647
Vasculature	vas	0.802	0.683	0.827	0.858	0.8	0.822	0.815	0.738
Mean of 16 Biodomains	avg	0.782	0.685	0.814	0.836	0.786	0.827	0.804	0.743

Supplementary Table 6. Predictive performance of applying unimodal and multimodal models trained on ROSMAP DLPFC training dataset to samples from ROSMAP DLPFC validation and MSBB PHG validation datasets. Prot: protein; Int: integrative. This data was used to generate Figure 5.

Biodomain Name	Biodomain Abbreviation	ROSMAP-RNA	MSBB-RNA	ROSMAP-Prot	MSBB-Prot	ROSMAP-Int	MSBB-Int
Apoptosis	apo	0.787	0.865	0.822	0.773	0.874	0.821
APP Metabolism	app	0.764	0.847	0.839	0.756	0.874	0.748
Autophagy	aut	0.769	0.841	0.791	0.719	0.84	0.75
Cell Cycle	cel	0.783	0.837	0.786	0.78	0.854	0.786
Endolysosome	end	0.793	0.804	0.828	0.741	0.871	0.76
Immune Response	imm	0.789	0.862	0.801	0.725	0.859	0.783
Lipid Metabolism	lip	0.802	0.863	0.81	0.757	0.842	0.768
Metal Binding	met	0.791	0.836	0.795	0.796	0.856	0.814
Mitochondrial Metabolism	mit	0.785	0.838	0.791	0.772	0.85	0.811
Myelination	mye	0.781	0.81	0.846	0.765	0.904	0.776
Oxidative Stress	oxi	0.777	0.781	0.788	0.754	0.847	0.77
Proteostasis	pro	0.79	0.864	0.805	0.759	0.854	0.821
Structural Stabilization	str	0.794	0.882	0.851	0.789	0.891	0.759
Synapse	syn	0.788	0.882	0.857	0.807	0.881	0.799
Tau Homeostasis	tau	0.717	0.712	0.832	0.731	0.877	0.761
Vasculature	vas	0.802	0.858	0.822	0.773	0.861	0.806

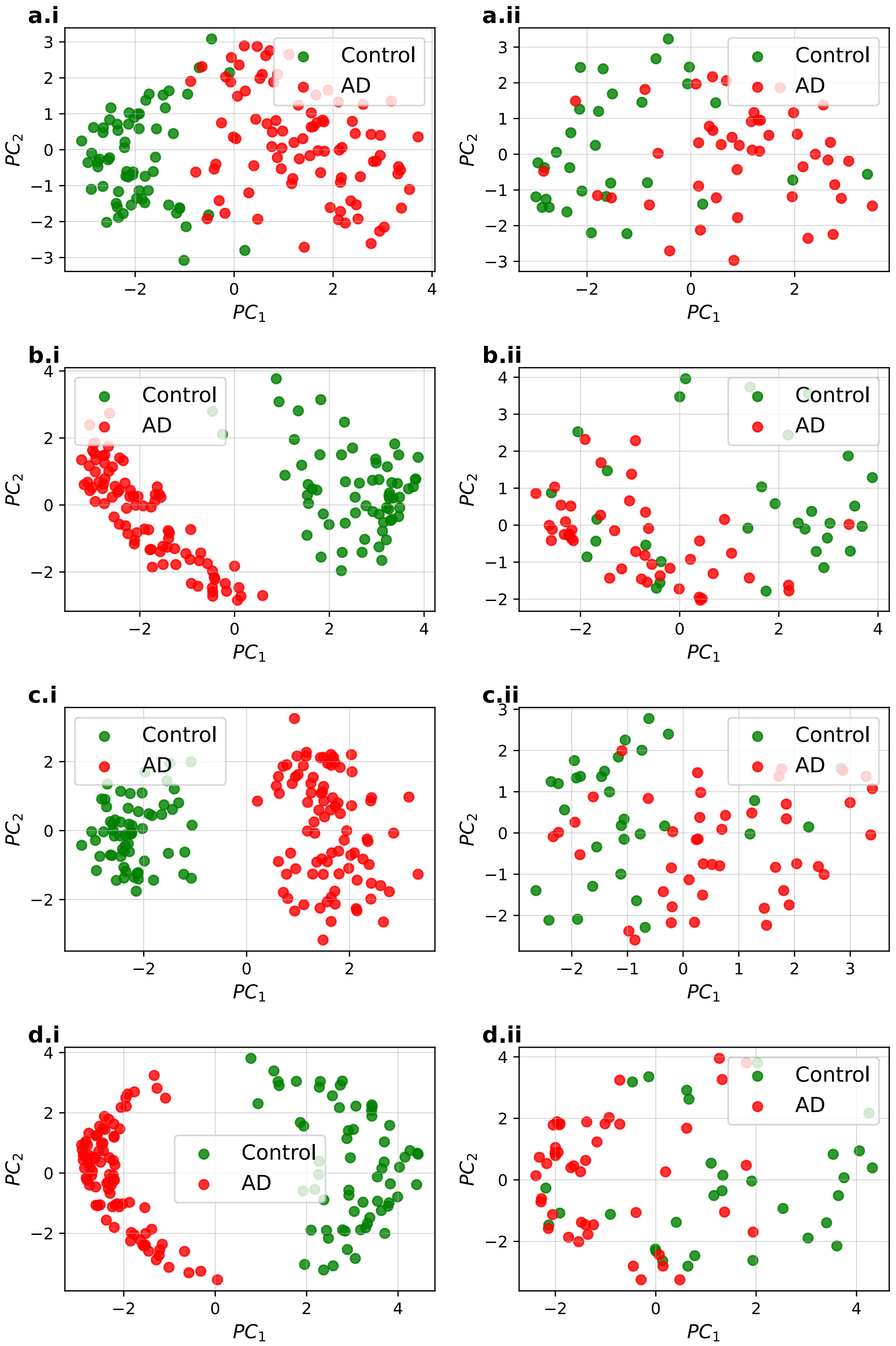

Supplementary Figure 1: Scatterplots of 1st and 2nd principal components of learned representations from our integrated model GNNRAI for four biodomains. Each row corresponds to a biodomain, with the left and right panels corresponding to the training and validation datasets respectively and denoted by the roman numerals ‘i’ and ‘ii’. From top to bottom – a. APP Metabolism, b. Endolysosome, c. Immune Response, and d. Synapse.
